# Psychometric properties of an Arabic translation of the Inflexible Eating Questionnaire (IEQ) in a non-clinical sample of adults

**DOI:** 10.1186/s40337-023-00835-7

**Published:** 2023-07-10

**Authors:** Feten Fekih-Romdhane, Vanessa Azzi, Diana Malaeb, Abir Sarray El Dine, Sahar Obeid, Souheil Hallit

**Affiliations:** 1 The Tunisian Center of Early Intervention in Psychosis, Department of Psychiatry “Ibn Omrane”, Razi Hospital, 2010 Manouba, Tunisia; 2 Faculty of Medicine of Tunis, Tunis El Manar University, Tunis, Tunisia; 3 School of Medicine and Medical Sciences, Holy Spirit University of Kaslik, P.O. Box 446, Jounieh, Lebanon; 4 School of Pharmacy, Lebanese International University, Beirut, Lebanon; 5 College of Pharmacy, Gulf Medical University, Ajman, United Arab Emirates; 6 Department of Biomedical Sciences, School of Arts and Sciences, Lebanese International University, Beirut, Lebanon; 7 School of Arts and Sciences, Social and Education Sciences Department, Lebanese American University, Jbeil, Lebanon; 8 Psychology Department, College of Humanities, Effat University, Jeddah, Saudi Arabia; 9 Applied Science Research Center, Applied Science Private University, Amman, Jordan; 10 Research Department, Psychiatric Hospital of the Cross, Jal Eddib, Lebanon

**Keywords:** Inflexible eating, Psychometrics, Test adaptation, Lebanon, Arabic

## Abstract

**Background:**

The Inflexible Eating Questionnaire (IEQ) is an 11-item instrument designed to evaluate the behavioural and psychological components of inflexible eating. However, the psychometric properties of the instrument have been infrequently examined, and no previous work has examined its utility in the context of the Middle East.

**Methods:**

A total of 826 Lebanese citizens and residents completed a novel Arabic translation of the IEQ, as well as previously validated measures of body appreciation, functionality appreciation, and disordered eating.

**Results:**

The unidimensional factor structure of the IEQ was upheld through both exploratory and confirmatory factor analyses, with all 11 items retained. We obtained evidence of scalar invariance across gender and found that there were no significant differences in observed IEQ scores between men and women. IEQ scores were also found to have adequate composite reliability and adequate patterns of concurrent validity.

**Conclusion:**

The present findings provide support for the psychometric properties of the Arabic version of the IEQ in examining inflexible eating in Arabic-speaking adults in Lebanon.

**Plain English Summary:**

Inflexible or rigid dietary restraint reflects an all-or-none approach that encompasses feeling compelled to obey a set of self-imposed dieting rules (e.g., avoiding high-calorie food, calorie counting, fasting to lose weight and/or skipping meals), having a sense of self-control and feeling empowered when adhering to these rules, and not respecting or following internal/external cues of hunger, satiety, and appetite. Therefore, the inflexible eating construct is composed of two dimensions, the first one is behavioural (i.e., obeying restrictive dietary rules) and the second one is psychological (i.e., the belief that following these rules is a consistent must). Until recently, the measures designed to assess inflexible eating focused on the behavioral dimension, while omitting to account for the psychological processes underlying the construct. To bridge this gap, the Inflexible Eating Questionnaire (IEQ), an 11-item self-report measure, was developed to assess both the behavioural and psychological components of dietary restraint. To date, the IEQ is not yet validated in Arabic. Through the present study, we aimed to examine the psychometric properties of an Arabic translation of the IEQ, which would in turn facilitate improved research and clinical practices related to dietary restraint in Arabic-speaking nations. Overall, findings provided support for the good psychometric qualities of the Arabic version of the IEQ, which suggests its utility for detecting inflexible eating in Arabic-speaking adults.

## Introduction

*Dietary restraint* (DR) is defined as the intention to control and reduce caloric intake deliberately in order to lose or maintain weight [1]. It is a complex and multifaceted concept that can have different outcomes [2] depending on whether it is flexible or rigid [3]. In contrast to flexible dietary approach (i.e., behaviours such as moderate taking of “healthy” or smaller servings of food to regulate weight), rigid dietary control refers to all-or-nothing approach to eating behaviours and attitudes toward dieting (e.g., inflexible dietary rules dictating how much, when, and what one should eat) [4, 5]. Rigid or inflexible DR encompasses two dimensions, namely behavioural (i.e., obeying restrictive rules that include calorie counting, avoiding high-calorie food, skipping meals, and even fasting to lose weight) [3], and psychological (i.e., the belief that one must constantly adhere to inflexible, arbitrary rules that provide a feeling of empowerment and foster a sense of self-control) [6]. Inflexible DR has been associated with detrimental health effects, including affective and emotional disturbances [7, 8], and body image concerns [4, 5, 9–11]. Moreover, there is evidence that inflexible eating is a significant predictor of disordered eating [7], and represents a critical aspect of understanding eating patterns and psychopathology [7, 12–14]. Therefore, investigating these dimensions separately would allow scholars to more effectively determine how the different facets of DR differently impact mental and physical health.

Several measurement instruments have been developed and are commonly used to assess DR, such as the Restraint Scale [1, 15], the Restraint subscale of the Eating Disorder Examination-Questionnaire (EDE-Q; [16], the Cognitive Restraint subscale of the Three Factor Eating Questionnaire [17], and the Dutch Restraint Eating Scale [18]. Some of these instruments have been validated across various countries and languages (e.g. [19],), but all are focused solely on the behavioural dimension (i.e., effort or attempts to restraint caloric consumption), and do not fully account for the psychological processes underlying inflexible dietary control [7]. To address this gap, Duarte et al. [7] developed the Inflexible Eating Questionnaire (IEQ), an 11-item instrument designed to evaluate the behavioural and psychological components of DR. Beginning with a pool of 25 items, an exploratory factor analysis (EFA) with a sample of Portuguese women allowed for the extraction of a unidimensional, 11-item model of IEQ scores. This unidimensional model was further supported in a second sample of Portuguese adults using confirmatory factor analysis (CFA) once the covariance of errors between two pairs of items was freed. This unidimensional model of IEQ scores was also shown to be fully invariant across women and men, to have adequate composite reliability, and good patterns of nomological validity (e.g., significant positive associations with scores on measures of eating psychopathology, body image inflexibility, general psychopathology, and intuitive eating).

To date, the psychometric properties of the IEQ have been examined in a small number of studies and linguistic versions, including Portuguese [20], English [21], Chinese [22], and German [23]. The instrument’s unidimensional factor structure has been supported using CFA in adolescents from Portugal [20] and China [22], as well as in women from Australia [21], and community adults from Germany and Austria [23]. These studies have also broadly supported additional psychometric indices, including invariance across boys and girls (Duarte et al. 2020; Tie et al. 2022), adequacy of composite reliability, and adequacy of the scale’s nomological network (see also [24]). Nevertheless, much more can be done to better understand the psychometric properties of the IEQ in diverse cultural and linguistic contexts, particularly as studies suggest that patterns of eating behaviours are likely to vary across such contexts (e.g., [19, 25, 26]. Arab cultures, in particular, may be a useful cultural context in which to further investigate the psychometric properties of the IEQ, given a historical preference for body shapes and size that diverge from appearance ideals common in the developed West [27, 28]. Additionally, Arab populations have historically been more likely to report greater physical inactivity [29], higher rates of obesity [30–32], lower body dissatisfaction [33], and less motivation to adopt thin appearance ideals [34] than non-Arab populations.

Nevertheless, there is also some evidence that contemporary Arab generations have become increasingly preoccupied with body shape and size, as Arab societies modernize and Westernize [35]. This has contributed to an increase in disordered eating psychopathology and its associated negative consequences in many Arab countries [35]. However, despite these data, literature on eating-related behaviours that has emerged from the Arab world still suffers from several major limitations due to the lack of validated and adapted screening instruments [35]. In particular, a recent literature review could only find seven studies focusing on restrained eating behaviour [35], with the Dutch Restrained Eating Scale being the only DR measure validated in Arabic in Lebanese adolescent [36] and adult [37] samples. Thus, it is imperative to perform further research on inflexible eating in different cultural backgrounds, including Arab cultures, to help validate the current hypothesized models of DR [21].

To that end, we aimed to translate and validate a novel Arabic version of the IEQ in the present study, which would in turn facilitate improved research and clinical practices related to DR in Arabic-speaking nations. To do so, we first examined the factorial validity of the IEQ. Given our expectation of a unidimensional model, we utilised an EFA-to-CFA strategy, as recommended in the literature [38]. This would allow us to first examine the factor structure of the IEQ without modelling limitations in our sample of Arabic-speaking Lebanese adults (i.e., through EFA), as well as to cross-validate the EFA-derived model of IEQ scores (i.e., through CFA). Additionally, we also expected to find evidence of scalar invariance of the IEQ across women and men, which would in turn allow us to examine gender differences in IEQ mean scores [39]. Finally, to assess broader psychometric properties of the IEQ in Lebanese adults, we examined associations with scores on theoretically plausible nomological constructs that have been validated for use in Arabic-speaking populations. Specifically, we expected that IEQ scores would be positively associated with disordered eating attitudes, and negatively associated with indices of positive body image (i.e., body appreciation and functionality appreciation).

## Methods

### Procedures

Data for the present data were collected as part of a larger project, portions of which have been reported on previously [40]. All data were collected via a Google Form link, between December 2021 and April 2022. The project was advertised on social media and included an estimated duration. Inclusion criteria for participation included being of a resident and citizen of Lebanon of adult age. Internet protocol (IP) addresses were examined to ensure that no participant took the survey more than once. After providing digital informed consent, participants were asked to complete the instruments described above, which were presented in a pre-randomised order to control for order effects. The survey was anonymous and participants completed the survey voluntarily and without remuneration.

### Participants

A total of 826 Lebanese citizens and residents enrolled in this study with a mean age of 25.42 years (*SD* = 8.44) and 57.9% women. Other sample characteristics are displayed in Table 1.

**Table 1:** Sociodemographic characteristics of the participants

Variable	Total sample (*N* = 826)	First split-half subsample (*n* = 411)	Second split-half subsample (*n* = 415)
Gender			
Men	348 (42.1%)	182 (44.3%)	166 (40.0%)
Women	478 (57.9%)	229 (55.7%)	249 (60.0%)
Education			
Primary	24 (2.9%)	14 (3.4%)	10 (2.4%)
Complementary	48 (5.8%)	30 (7.3%)	18 (4.3%)
Secondary	123 (14.9%)	63 (15.3%)	60 (14.5%)
University	631 (76.4%)	304 (74.0%)	327 (78.8%)

	Mean ± SD		
Age (in years)	25.42 ± 8.44	25.86 ± 9.18	24.97 ± 7.63
Body mass index (kg/m^2^)	23.81 ± 4.83	23.82 ± 5.00	23.80 ± 4.67

### Measures

#### Inflexible Eating Questionnaire

The IEQ (Duarte et al. 2017) is composed of 11 items rated on a 5-point Likert-type scale from 1 (*fully disagree*) to 5 (*fully agree*), with higher scores reflecting greater inflexibility. To translate the IEQ into Arabic, we used a combination of methods, as per test adaptation recommendations [38]. First, the IEQ was translated from English into Arabic by a bilingual translator, whose native language is Arabic and who is fluent in English. Next, an expert committee formed by healthcare professionals and a linguistics expert verified the translated Arabic version of the scale. Subsequently, the Arabic version of the scale was back-translated into English by an independent translator who is fluent in Arabic and English. The back-translated measure was returned to the expert committee, who compared both translations and aimed to resolve any inconsistencies between versions. This process was repeated until all minor ambiguities had been resolved. The IEQ items in English are reported in Table 1 and the items in Arabic are reported in Appendix 1.

#### Functionality appreciation

We used the Functionality Appreciation Scale (FAS; [41]; Arabic translation: [40]), a 7-item measure of participants’ appreciation of what the body does and can do. All items were rated on a 5-point scale, ranging from 1 (*strongly disagree*) to 5 (*strongly agree*). An overall score was computed as the mean of all items, with higher scores reflecting greater functionality appreciation. Scores on the Arabic version of the FAS have been reported to have a unidimensional factor structure, adequate composite reliability, and adequate construct validity Swami et al. [40]. In the present study, McDonald’s ω for FAS scores was 0.96 (95% CI = 0.95, 0.97) in the total sample.

#### Body appreciation

Participants were asked to complete the Body Appreciation Scale-2 (BAS-2; [42]; Arabic translation: Vally et al. [43]). This 10-item instrument assesses acceptance of one’s body, respect and care for one’s body, and protection of one’s body from unrealistic beauty standards. All items were rated on a 5-point scale, ranging from 1 (*never*) to 5 (*always*), and an overall score was computed as the mean of all items. Higher scores on this scale reflect greater body appreciation. Scores on the Arabic version of the BAS-2 have been shown to have a unidimensional factor structure, adequate composite reliability, and adequate convergent validity [43]. In the present study, McDonald’s ω for BAS-2 scores was 0.97 (95% CI 0.96, 0.97) in the total sample.

#### Disordered eating

Participants were asked to complete the short form of the Eating Attitudes Test-7 (EAT-7; [44]). This 7-item measures symptoms and concerns characteristic of eating disorders. All items were rated on a 6-point scale, ranging from 1 (*never*) to 6 (*always*). Higher total scores reflect greater disordered eating attitudes. In the present study, McDonald’s ω for overall EAT-7 scores was 0.90 (95% CI 0.89, 0.91) in the total sample.

#### Demographics

Participants were asked to provide their demographic details consisting of age, gender, highest educational attainment, height, and weight. Height and weight data were used to compute self-reported body mass index (BMI) as kg/m^2^.

### Analytic strategy

#### Data treatment

There were no missing responses in the dataset. To examine the factor structure of the IEQ, we used an EFA-to-CFA strategy [38]. To ensure adequate sample sizes for both EFA and CFA, we split the main sample using an SPSS computer-generated random technique; sample characteristics of the two split-halves are reported in Table 1. There were no significant differences between the two subsamples in terms of mean age, *t*(824) = 1.52, *p* = 0.129, *d* = 0.11, BMI, *t*(824) = 0.07, *p* = 0.944, *d* = 0.004, and the distribution of women and men, χ^2^(1) = 1.55, *p* = 0.213.

#### Exploratory factor analysis

To explore the factor structure of IEQ, we computed a principal-axis EFA with the first split-half subsample using the FACTOR software [45, 46]. We verified all requirements related to item-communality (≥ 0.3) ([47], average item correlations (0.15–0.50), and item-total correlations (≥ 0.15) [48]. The Kaiser–Meyer–Olkin (KMO) measure of sampling adequacy (which should ideally be ≥ 0.80) and Bartlett’s test of sphericity (which should be significant) ensured the adequacy of our sample [49]. The procedure for determining the number of factors to extract was parallel analysis (PA; [50] using the Pearson correlation matrix. Weighted Root Mean Square Residual (WRMR) was also calculated to assess the model fit (values < 1 have been recommended to represent good fit; [51].

Item retention was based on the recommendation that items with “fair” loadings and above (i.e., ≥ 0.33) and with low inter-item correlations (suggestive of low item redundancy) as indicated by the anti-image correlation matrix should be retained (≥ 0.50) [52]. Two EFAs were done separately on males and females respectively.

#### Confirmatory factor analysis

We used data from the second split-half to conduct a CFA using the SPSS AMOS v.26 software. Proactive Monte Carlo simulations [53] indicated that a sample size of 202 would be sufficient for this analysis, which was surpassed in our study. Our intention was to test the parent model of IEQ scores (i.e., a unidimensional model; [7] and, if divergent, any models extracted from our EFA. The analysis was done using the maximum likelihood estimation method. We considered adding a correlation between residuals of items in case the modification indices were high. Additionally, evidence of convergent validity was assessed in this subsample using the average variance extracted (AVE), with values of ≥ 0.50 considered adequate [54].

#### Gender invariance

To examine gender invariance of IEQ scores, we conducted multi-group CFA [39] using the second split-half subsample. Measurement invariance was assessed at the configural, metric, and scalar levels [55]. Configural invariance implies that the latent IEQ variable(s) and the pattern of loadings of the latent variable(s) on indicators are similar across gender (i.e., the unconstrained latent model should fit the data well in both groups). Metric invariance implies that the magnitude of the loadings is similar across gender; this is tested by comparing two nested models consisting of a baseline model and an invariance model. Lastly, scalar invariance implies that both the item loadings and item intercepts are similar across gender and is examined using the same nested-model comparison strategy as with metric invariance [39]. Following previous recommendations [39, 56], we accepted ΔCFI ≤ 0.010 and ΔRMSEA ≤ 0.015 or ΔSRMR ≤ 0.010 (0.030 for factorial invariance) as evidence of invariance. We aimed to test for gender differences on latent IEQ scores using an independent-samples *t*-test only if scalar or partial scalar invariance were established.

#### Further analyses

Composite reliability in both subsamples was assessed using McDonald’s ω and its associated 95% CI, with values greater than 0.70 reflecting adequate composite reliability [57]. McDonald’s ω was selected as a measure of composite reliability because of known problems with the use of Cronbach’s α [58]. To assess convergent and criterion-related validity, we examined bivariate correlations between IEQ scores and those on the additional measures included in the survey (functionality appreciation, body appreciation, and disordered eating attitudes) using the total sample. All scores had normal distribution, as identified by skewness and kurtosis values varying between − 1 and + 1 [59]; therefore, Pearson correlation test was used. Based on [60], values ≤ 0.10 were considered weak, ~ 0.30 were considered moderate, and ~ 0.50 were considered strong correlations.

## Results

### Exploratory factor analysis

#### Factor analysis with women

For women from the first split-half subsample, Bartlett’s test of sphericity, χ^2^(55) = 1325.2, *p* < 0.001, and KMO (0.92) indicated that the IEQ items had adequate common variance for factor analysis. The results of the EFA revealed one factor, which explained 54.40% of the common variance (item-factor loadings ≥ 0.65). The WRMR value was also adequate (0.071), indicating good fit of the model.

#### Factor analysis with men

For men from the first split-half subsample, Bartlett’s test of sphericity, χ^2^(55) = 1149.4, *p* < 0.001, and KMO (0.90) again indicated that the IEQ items had adequate common variance for factor analysis. The results of the EFA revealed one factor, which explained 55.98% of the common variance (item-factor loadings ≥ 0.64). The WRMR value was also adequate (= 0.065), indicating good fit of the model.

#### Factor structure congruence and composite reliability

The factor loadings reported in Table 2 for women and men separately suggest strong similarity across factor structures. Tucker’s congruence coefficient was 0.97, suggesting that the factor structures were equal in women and men. McDonald’s ω was adequate in women (ω = 0.91, 95% CI 0.89, 0.93), men (ω = 0.91, 95% CI 0.89, 0.93), and the total subsample (ω = 0.91, 95% CI 0.89, 0.93).

**Table 2:** Items of the Inflexible Eating Questionnaire in English and Factor Loadings Derived from the Exploratory Factor Analyses (EFA) with Women and Men in the First Split-Half Subsample, and Standardised Estimates of Factor Loadings from the Confirmatory Factor Analysis (CFA) in the Second Split-Half Subsample

Item	EFA		CFA
	Women	Men	Total
(1) When I cannot follow my eating plan, I feel very anxious (or nervous)	0.75	0.74	0.74
(2) When I do not follow one of my eating rules, then I make an effort to compensate it by following my rules even more strictly	0.74	0.78	0.72
(3) For me, having a balanced eating pattern requires strictly following certain rules	0.66	0.77	0.69
(4) Having well-defined eating rules makes me feel organized/in control	0.74	0.72	0.68
(5) I would rather follow my eating rules than to eat without any guidance or according to my appetite or will	0.65	0.64	0.59
(6) If I notice any change in my weight (even a small one), following my diet becomes a priority for me	0.72	0.76	0.79
(7) I get worried when I do not follow my eating rules, even if it only happens occasionally	0.79	0.82	0.78
(8) Even if I feel satisfied with my weight, I do not allow myself to ease my eating rules	0.71	0.76	0.70
(9) I feel proud when I can rigidly follow certain eating rules	0.77	0.71	0.70
(10) Not following my eating rules makes me feel inferior	0.78	0.71	0.71
(11) To manage my eating through rules gives me a sense of control	0.79	0.80	0.73

### Confirmatory factor analysis

CFA with the second split-half subsample indicated that fit of the unidimensional model of IEQ scores was generally acceptable: χ^2^/*df* = 182.35/44 = 4.14, RMSEA = 0.087 (90% CI 0.074, 0.100), SRMR = 0.037, CFI = 0.942, TLI = 0.927. When adding a correlation between residuals for items #9 and 11, fit indices were improved and uniformly acceptable: χ2/*df* = 115.29/43 = 2.68, RMSEA = 0.064 (90% CI 0.050, 0.078), SRMR = 0.030, CFI = 0.970, TLI = 0.961. The standardised estimates of factor loadings were all adequate (see Table 1). The convergent validity for this model was adequate, as AVE = 0.51.

#### Composite reliability

Composite reliability of scores was adequate in women (ω = 0.91, 95% CI 0.88, 0.94), men (ω = 0.93, 95% CI 0.90, 0.96), and the total sample (ω = 0.92, 95% CI 0.89, 0.95).

### Gender invariance

Next, we tested for gender invariance based on the unidimensional model of IEQ scores in the second split-half subsample. As reported in Table 3, all indices suggested that configural, metric, and scalar invariance was supported across gender. Given these results, we computed an independent-samples *t*-test to examine gender differences in IEQ scores. The results showed that no significant difference was found in terms of IEQ scores between women (*M* = 32.19, *SD* = 9.52) and men (*M* = 33.12, *SD* = 9.08) in the second subsample, *t*(413) = 1.01, *p* = 0.315, *d* = 0.10.

**Table 3:** Measurement invariance across gender in the second split-half subsample

Model	χ^2^	*Df*	CFI	RMSEA	SRMR	Model comparison	Δχ^2^	ΔCFI	ΔRMSEA	ΔSRMR	Δ*df*	*p*
Configural	330.30	86	0.947	0.059	0.038							
Metric	340.95	97	0.947	0.055	0.042	Configural vs metric	10.65	< 0.001	0.004	0.004	11	0.473
Scalar	350.69	96	0.944	0.057	0.032	Metric vs scalar	20.39	0.003	0.002	0.006	10	0.025

CFI = Comparative fit index; RMSEA = Steiger-Lind root mean square error of approximation; SRMR = Standardised root mean square residual.

### Convergent and criterion-related validity

To assess the validity of IEQ scores, we examined bivariate correlations with all other measures included in the present study separately for women and men using the total sample. Inflexible eating was negatively and significantly correlated with body appreciation, and functionality appreciation in both men (Table 4) and women (Table 5). Inflexible eating was significantly and moderately associated with higher EAT scores in men, but not women. For exploratory purposes, we also examined associations with age and BMI. Inflexible eating was not significantly associated with age in men (*r* = 0.03, *p* = 0.620) and women (*r* = − 0.01; *p* = 0.839), but was significantly associated with lower BMI in both men (*r* = − 0.24, *p* < 0.001) and women (*r* = − 0.13, *p* = 0.004).

**Table 4:** Bivariate correlations between Inflexible Eating Scores on other measures included in the study in men

	Mean ± SD	(1)	(2)	(3)	(4)
(1) Inflexible eating	32.21 ± 8.80	1			
(2) Disordered eating attitudes	5.18 ± 6.10	0.25***	1		
(3) Body appreciation	34.54 ± 10.09	− 0.40***	− 0.57***	1	
(4) Functionality appreciation	24.27 ± 7.00	− 0.42***	− 0.55***	0.83***	1

***p < 0.001

**Table 5:** Bivariate correlations between Inflexible Eating Scores on other measures included in the study in women

	Mean ± SD	(1)	(2)	(3)	(4)
(1) Inflexible eating	33.36 ± 8.83	1			
(2) Disordered eating attitudes	4.38 ± 4.97	0.07	1		
(3) Body appreciation	38.11 ± 9.06	− 0.15**	− 0.30***	1	
(4) Functionality appreciation	26.91 ± 5.56	− 0.19***	− 0.44***	0.66***	1

*p < 0.05, **p < 0.01, ***p < 0.001

## Discussion

The goal of the current research was to investigate the psychometric properties of the IEQ, translated into Arabic, in a community sample of Lebanese adults. Our findings supported a unidimensional factor structure of IEQ scores across both split-half subsamples (i.e., using both EFA and CFA). This unidimensional model of IEQ scores was invariant across gender, consistently demonstrated adequate composite reliability, and showed expected concurrent validity. In broad outline, these results indicate that the Arabic IEQ is a valid and reliable instrument to capture the construct of inflexible eating in Arabic-speaking populations, at least in Lebanon.

As expected, the present study supported a unidimensional model of IEQ scores, which is consistent with analyses in adolescents [22, 61] and adults [7, 21] in other national contexts. More specifically, the results of both our EFA and CFA supported a unidimensional model of IEQ scores, with all 11 items retained. However, the CFA indicated that it was necessary to free the error covariance between one pair of items (i.e., Items #9 and 11) in order to achieve optimal fit. This is consistent with the CFA findings in the parent study (Duarte et al. 2017), although item pairs for which covariances were freed differed. In the present study, it is possible that Items #9 and 11 share some similarity of meaning, given that both refer to emotional states that result from following rigid eating rules (i.e., pride and a sense of control, respectively). Given that freeing error covariances may be construed as a form of exploratory modelling, it may be useful in future work to further assess this aspect of our results. More broadly, given the need to free error covariances across studies, there may be value in determining whether the IEQ could be truncated further without substantive loss of meaning.

Beyond factor structure, our findings also suggested that the IEQ in its Arabic version has adequate composite reliability, which is consistent with previous work [7, 21, 22]. Additionally, multigroup CFA was conducted to test the measurement invariance of the IEQ with respect to gender. Our results showed that the unidimensional model of IEQ scores achieved scalar invariance. This is particularly notable because scalar invariance has been demonstrated previously in adolescent samples [22, 61], but has not been demonstrated in adults beyond the parent study [7]. Establishing scalar invariance also allowed us to compare observed IEQ scores across gender groups. Our results indicated that there was no gender difference in inflexible eating. Previously, in an adult Portuguese sample, Duarte et al. [7] reported that women had significantly higher inflexible eating scores compared to men. However, the (unreported) effect size of the difference was small-to-medium (*d* = 0.29), which suggests that any gender difference may not have much real-world practical value. Nevertheless, it was also notable that other work in the Lebanese context has indicated that women tend to score more highly than men on measures of DR (e.g., [36, 37]. As such, it may be useful to investigate gender differences in inflexible eating further in more representative samples of Lebanese adults.

Additionally, the results of our study broadly supported the concurrent validity of IEQ scores. Specifically, we found that inflexible eating was significantly and negatively associated with indices of positive body image (i.e., body appreciation and functionality appreciation), although effect sizes of these relationships were substantially stronger in men (medium effects) than women (weak effects). On the other hand, associations between inflexible eating and disordered eating attitudes were more equivocal, with the relationship being significant and positive in men, but not reaching significance in women. Given that the EAT-7 should gender invariance in the original study [44], the results obtained in this current paper might be due to the floor effect seen in women as 149 women (31.2%) scored 0 on the EAT scale. More investigation is further needed in future research.

### Study limitations

Although the present findings are important in terms of better understanding the cross-national utility of the IEQ, they should be interpreted in light of certain limitations. First, the present data only included adult participants from the community, making any assumptions regarding people of different age groups (e.g., adolescents) or settings (e.g., patients with eating disorders or with a body mass index ≥ 30) not presently possible. Further validations in clinical populations and of different age groups could usefully extend our findings. Second, while the Arabic IEQ has proven to be valid and reliable when used in a Lebanese Arabic-speaking population, it may not be sensitive in capturing cross-cultural differences found between various Arab communities [35] and thus still requires validation in other Arab countries and cultures to confirm its psychometric validity. Third, it was not within the scope of the current study to investigate the discriminant validity and test–retest reliability of the Arabic IEQ; therefore, exploring these psychometric properties remains subjects for future research.

## Conclusion

Altogether, the present findings provide support for the validity and usefulness of the Arabic version of the 11-item IEQ in examining inflexible eating in Arabic-speaking adults in Lebanon. We hope that making this instrument available for clinicians and researchers from Arab countries would enable a deeper examination of the specific clinical features, risk factors, and evolution patterns of inflexible eating, that may be attributable to influence by the local cultural context. The Arabic IEQ would also allow for extended research and cross-cultural comparisons to be conducted in the still under-researched field of disordered eating in the Arab world.

## Data Availability

The authors do not have the right to share any data information as per their institutions policies (copyright issues). Data can be shared upon a reasonable request to the corresponding author (S.H.).
